# Supplementary material for: Unravelling the Effects of the Healthy Primary School of the Future: For Whom and Where Is It Effective?
Source: Nutrients. 2019 Sep 5;11(9):2119. doi: 10.3390/nu11092119 (PMC6770282; doi:10.3390/nu11092119)
Supplement: Supplementary file 1 [file nutrients-11-02119-s001.pdf]

<u>**Supplementary file 1. Clustering of parenting practices**</u>

Clustering of nutrition-related practices of parents

Preparation of the data

Before running cluster analyses, parents with missing data on the practices were excluded (n=0), univariate outliers were replaced by the mean score plus three standard deviations (0.83% of all responses on FPPs), and multivariate outliers (15 cases) were eliminated from the further analyses.

The four clusters

Cluster 1 (n = 226; 36.9%), labelled "*high involvement and supportive*", was characterized by relatively high scores on most types of nutrition-related parenting practices.

Cluster 2 (n = 102; 16.7%), labelled "*low covert control and non-rewarding*", consisted of parents with relatively moderate scores on most practices, but with lower scores on accessibility of healthy foods and low use of emotional feeding.

Cluster 3 (n = 78; 12.7%), labelled "*low involvement and indulgent*", had relatively low scores on most types of nutrition-related parenting practices.

Cluster 4 (n = 206; 33.7%), labelled "*high covert control and rewarding*", was characterized by relatively moderate scores on most practices, but with higher scores on accessibility of healthy foods and high use of emotional feeding.

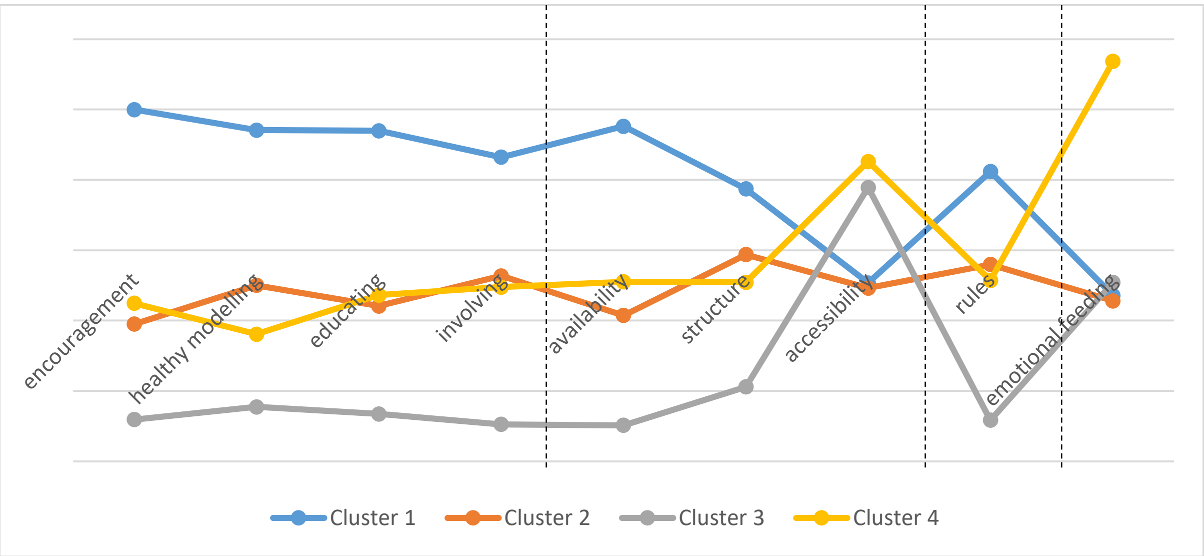

Figure S1. Graphical view of the four-cluster solution based on mean z-scores for all nutrition-related parenting practices (n=612)

Higher scores indicate more frequent use of the FPP; Each of the successive graphical areas represents a distinct category of nutrition-related parenting practices, i.e., *responsiveness, structure, behavioural control and psychological control; Cluster 1 "high involvement and supportive": blue line; Cluster 2 "low covert control and non-rewarding": orange line; Cluster 3 "low involvement and indulgent": grey line; Cluster 4 "high covert control and rewarding": yellow line.*

Clustering of PA-related practices of parents

Preparation of the data

Before running cluster analyses, parents with missing data on the practices were excluded (n=5), univariate outliers were replaced by the mean score plus three standard deviations (0.68% of all responses on FPPs), and multivariate outliers (16 cases) were eliminated from the further analyses.

Exploration of the most optimal clustering

Since the clustering of Gevers et al. focused only on nutrition-related parenting practices, we conducted the full exploration of the most optimal clustering. Ward's method indicated that a four-cluster solution gave the best fit, in view of the change in agglomeration coefficients. After considering this outcome and the dendrogram, we conducted k-means cluster analyses using 3- and 4-cluster solutions, from which a four-cluster solution was derived. After replicating the full two-step clustering approach in two subsamples, we obtained a Cohen's kappa of 0.814, indicating substantial stability of the cluster solution.

The four clusters

Cluster 1 (n = 220; 35.0%), labelled "*high involvement and supportive*", was characterized by relatively high scores on most types of PA-related parenting practices.

Cluster 2 (n = 133; 21.2%), labelled "*moderate involvement, indulgent of child's sedentary activities*", was characterized by relatively moderate scores on most practices, but with lower scores on sedentary-related practices.

Cluster 3 (n = 17; 2.7%), labelled "*low involvement and indulgent*", had relatively low scores on most types of PA-related parenting practices.

Cluster 4 (n = 258; 41.1%), labelled "*moderate involvement, supportive of child's sedentary activities*", was characterized by relatively moderate scores on most practices, but with higher scores on sedentary-related practices.

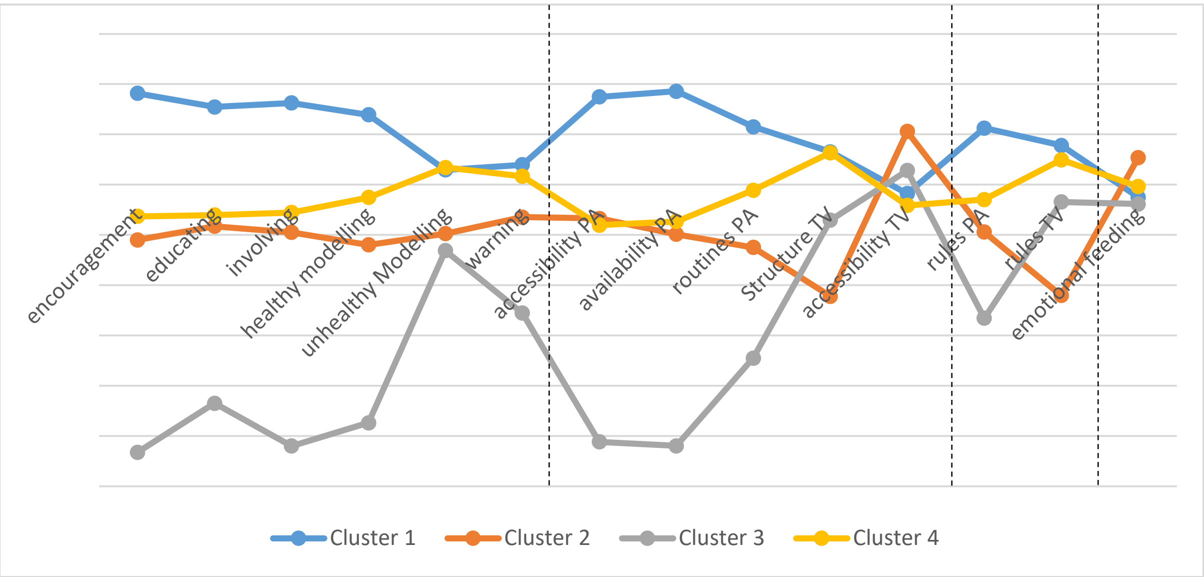

Figure S2. Graphical view of the four-cluster solution based on mean z-scores for all PA-related parenting practices (n=628)

Higher scores indicate more frequent use of the PA-PP; Each of the successive graphical areas represents a distinct category of PA-related parenting practices, i.e., *responsiveness, structure, behavioural control and psychological control. Cluster 1 “high involvement and supportive”: blue line; Cluster 2 “moderate involvement, indulgent of child’s sedentary activities”: orange line; Cluster 3 “low involvement and indulgent”: grey line; Cluster 4 “moderate involvement, supportive of child’s sedentary activities”: yellow line.*

Supplementary file 2 Characteristics of study sample

Table S1. Characteristics of study sample at baseline (T0), derived from Bartelink et al. (18)

		Total		Full HPSF [a]		Partial HPSF [b]		Control [c]		Chi-square / F-value	p-value
		N [d]	% / Mean (±SD)	N	% / Mean (±SD)	N	% / Mean (±SD)	N	% / Mean (±SD)		
**Gender (% boys)**		1676	47.4%	537	47.7%	478	47.3%	661	47.2%	.029 [f]	.986
**Age (years)**		1676	7.5 (±2.16)	537	7.6 (±2.16)	478	7.4 (±2.22)	661	7.6 (±2.13)	1.610	.200
**Study year [e]**		1676	4.0 (±2.00)	537	4.0 (±2.00)	478	3.8 (±2.01)	661	4.1 (±1.99)	2.526	.080
**Ethnicity (% Western)**		1016	94.1%	341	93.0%	326	96.0%	349	93.4%	3.239 [f]	.198
**SES**	*Lowest tertile*	1117	32.6%	361	28.8%	365	32.3%	391	36.3%	5.636 [f]	.228
**(%)**	*Middle tertile*		34.0%		35.7%		35.6%		30.9%		
	Highest tertile		33.4%		35.5%		32.1%		32.7%		
**BMI z-score**		1109	.135 (±1.02)	321	.051 (±1.01)	352	.092 (±.95)	436	.232 (±1.07)	3.399	**.034**
**Overweight/ obese (%)**		1109	19.9%	321	16.5%	352	17.9%	436	24.1%	14.156 [f]	**.006**

[a] Sample size of the number of children included in the analyses (N) and response rate at baseline (%) of the two full HPSF schools: S1: N=301, 68.0%; S2: N=236, 68.3%.

[b] Sample size of the number of children included in the analyses (N) and response rate at baseline (%) of the two partial HPSF schools: S3: N=190, 69.5%; S4: N=288, 68.0%.

[c] Sample size of the number of children included in the analyses (N) and response rate at baseline (%) of the four control schools: S5: N=142, 46.1%; S6: N=233, 51.4%; S7: N=193, 51.5%; S8: N=93, 55.8%.

[d] Observed N, missing data was due to later participation in the study, incomplete parent questionnaire, or because no height/weight was measured in study year 1.

[e] Study year 1-8 in Dutch system is comparable to two years of kindergarten followed by grade 1-6.

[f] Chi-square test.

Bold p-value = significant (<.05) difference

Abbreviations: SD = standard deviation; C.I. = confidence interval; ES = Effect size.

Supplementary file 3 Pairwise comparisons of the effect modifiers of HPSF

Table S2a. Pairwise comparisons of the effect modifiers of HPSF on the overall intervention effects

	Full HPSF vs. control		Partial HPSF vs. control	
	**B (95% C.I.)**	**p**	**B (95% C.I.)**	**p**
**SES**				
**Sedentary (% per day)**				
Lowest tertile vs highest tertile	.63 (-2.30 - 3.57)	.67	.94 (-1.99 - 3.87)	.53
Middle tertile vs highest tertile	.93 (-1.91 - 3.77)	.52	-.38 (-3.33 - 2.56)	.80
Lowest tertile vs middle tertile	-.30 (-3.50 - 2.91)	.86	1.32 (-1.75 - 4.40)	.40
**Light PA (% per day)**				
Lowest tertile vs highest tertile	-.41 (-2.73 - 1.90)	.73	-.20 (-2.58 - 2.18)	.87
Middle tertile vs highest tertile	-.65 (-2.93 - 1.62)	.57	-.13 (-2.51 - 2.25)	.91
Lowest tertile vs middle tertile	.24 (-2.27 - 2.76)	.85	-.07 (-2.55 - 2.42)	.96
**MVPA (% per day)**				
Lowest tertile vs highest tertile	-.21 (-1.41 - 1.00)	.74	-.72 (-1.89 - .45)	.23
Middle tertile vs highest tertile	-.25 (-1.40 - .90)	.67	.51 (-.63 - 1.65)	.38
Lowest tertile vs middle tertile	.05 (-1.25 - 1.34)	.95	-1.23 (-2.44 - -.03)	.05
**Healthy dietary behaviours (mean days/week)**				
Lowest tertile vs highest tertile	.13 (-.32 - .58)	.56	.18 (-.26 - .61)	.43
Middle tertile vs highest tertile	.25 (-.19 - .68)	.26	.09 (-.35 - .53)	.70
Lowest tertile vs middle tertile	-.11 (-.60 - .37)	.65	.09 (-.37 - .55)	.71
**Unhealthy dietary behaviours (mean days/week)**				
Lowest tertile vs highest tertile	.26 (-.09 - .62)	.15	.28 (-.07 - .63)	.11
Middle tertile vs highest tertile	.26 (-.08 - .60)	.13	.47 (.12 - .82)	.01
Lowest tertile vs middle tertile	.00 (-.37 - .38)	.99	-.19 (-.55 - .17)	.30
**PA-related parenting practices**				
**Sedentary (% per day)**				
Cluster 2 vs Cluster 1	.66 (-2.92 - 4.25)	.72	.63 (-2.99 - 4.26)	.73
Cluster 3 vs Cluster 1	2.93 (-2.15 - 8.01)	.26	1.42 (-4.17 - 7.01)	.62
Cluster 4 vs Cluster 1	1.24 (-2.02 - 4.50)	.45	.28 (-2.79 - 3.36)	.86
Cluster 3 vs Cluster 2	2.27 (-3.08 - 7.62)	.40	.78 (-5.11 - 6.68)	.79
Cluster 4 vs Cluster 2	.58 (-2.86 - 4.02)	.74	-.35 (-3.83 - 3.13)	.84
Cluster 4 vs Cluster 3	-1.69 (-6.70 - 3.32)	.51	-1.13 (-6.63 - 4.36)	.68
**Light PA (% per day)**				
Cluster 2 vs Cluster 1	-.31 (-3.15 - 2.54)	.83	.36 (-2.53 - 3.24)	.81
Cluster 3 vs Cluster 1	-1.71 (-5.75 - 2.33)	.41	-.66 (-5.27 - 3.95)	.78
Cluster 4 vs Cluster 1	-.66 (-3.21 - 1.89)	.61	-.07 (-2.52 - 2.39)	.96
Cluster 3 vs Cluster 2	.25 (-2.73 - 3.23)	.87	-1.40 (-5.80 - 3.00)	.53
Cluster 4 vs Cluster 2	.58 (-1.36 - 2.52)	.56	-.35 (-3.06 - 2.35)	.80
Cluster 4 vs Cluster 3	1.05 (-3.04 - 5.14)	.61	.59 (-4.03 - 5.21)	.80
**MVPA (% per day)**				
Cluster 2 vs Cluster 1	-.34 (-1.79 - 1.10)	.64	-.97 (-2.45 - .51)	.20
Cluster 3 vs Cluster 1	-1.23 (-3.55 - 1.10)	.30	-.72 (-2.92 - 1.49)	.52
Cluster 4 vs Cluster 1	-.58 (-1.93 - .77)	.40	-.24 (-1.52 - 1.03)	.71
Cluster 3 vs Cluster 2	-.88 (-3.08 - 1.32)	.43	.25 (-2.02 - 2.53)	.83
Cluster 4 vs Cluster 2	-.24 (-1.63 - 1.16)	.74	.73 (-.77 - 2.22)	.34
Cluster 4 vs Cluster 3	.65 (-1.50 - 2.79)	.55	.47 (-1.66 - 2.60)	.66
**Nutrition-related parenting practices**				
**Healthy dietary behaviours (mean days/week)**				
Cluster 2 vs Cluster 1	-.07 (-.55 - .41)	.76	.02 (-.44 - .49)	.93
Cluster 3 vs Cluster 1	.18 (-.48 - .83)	.60	.08 (-.58 - .74)	.81
Cluster 4 vs Cluster 1	-.09 (-.61 - .43)	.73	-.08 (-.63 - .47)	.78
Cluster 3 vs Cluster 2	.25 (-.41 - .91)	.46	.06 (-.59 - .71)	.86
Cluster 4 vs Cluster 2	-.02 (-.54 - .51)	.95	-.10 (-.64 - .44)	.71
Cluster 4 vs Cluster 3	-.27 (-.95 - .42)	.45	-.16 (-.87 - .55)	.66
**Unhealthy dietary behaviours (mean days/week)**				
Cluster 2 vs Cluster 1	.03 (-.35 - .41)	.87	.32 (-.07 - .71)	.10
Cluster 3 vs Cluster 1	.11 (-.42 - .64)	.69	.38 (-.17 - .92)	.17
Cluster 4 vs Cluster 1	.01 (-.41 - .43)	.96	.34 (-.10 - .78)	.13
Cluster 3 vs Cluster 2	.08 (-.45 - .60)	.77	.06 (-.48 - .59)	.84
Cluster 4 vs Cluster 2	-.02 (-.44 - .40)	.93	.02 (-.41 - .45)	.94
Cluster 4 vs Cluster 3	-.10 (-.65 - .46)	.73	-.04 (-.60 - .53)	.90

Significance level for the interaction-term: p<0.10.
Clusters nutrition-related parenting practices: Cluster 1 "high involvement and supportive"; Cluster 2 "low covert control and non-rewarding"; Cluster 3 "low involvement and indulgent"; Cluster 4 "high covert control and rewarding".
Clusters PA-related parenting practices: Cluster 1 "high involvement and supportive"; Cluster 2 "moderate involvement, indulgent of child's sedentary activities"; Cluster 3 "low involvement and indulgent"; Cluster 4 "moderate involvement, supportive of child's sedentary activities".
Abbreviations: HPSF: Healthy primary School of the Future; CI: confidence interval; p: p-value; ES: effect size; PA: physical activity; MVPA: moderate-to-vigorous physical activity; na: not applicable.

Table S2b. Pairwise comparisons of the effect modifiers of HPSF on intervention effects at school

	Full HPSF vs. control		Partial HPSF vs. control	
	**B (95% C.I.)**	**P**	**B (95% C.I.)**	**p**
**SES**				
**Sedentary (% per day at school)**				
Lowest tertile vs highest tertile	.40 (-1.40 - 2.19)	.83	.27 (-.79 - 1.34)	.87
Middle tertile vs highest tertile	-1.03 (-2.79 - .74)	.56	-.24 (-3.59 - 3.10)	.89
Lowest tertile vs middle tertile	1.42 (-1.80 - 4.65)	.44	.52 (-1.26 - 2.30)	.77
**Light PA (% per day at school)**				
Lowest tertile vs highest tertile	-.58 (-3.53 - 2.37)	.71	.40 (-2.48 - 3.29)	.79
Middle tertile vs highest tertile	.15 (-2.76 - 3.07)	.92	.05 (-2.85 - 2.94)	.98
Lowest tertile vs middle tertile	-.73 (-3.74 - 2.28)	.64	.36 (-2.48 - 3.20)	.81
**MVPA (% per day at school)**				
Lowest tertile vs highest tertile	.18 (-.91 - 1.26)	.75	-.65 (-1.72 - .41)	.23
Middle tertile vs highest tertile	.87 (-.21 - 1.94)	.12	.20 (-.87 - 1.27)	.71
Lowest tertile vs middle tertile	-.69 (-1.81 - .44)	.23	-.85 (-1.94 - .24)	.12
**Minimal two food types during lunch * (% yes)**				
Lowest tertile vs highest tertile	.74 (.14 - 4.02)	.73	.83 (.17 - 3.93)	.81
Middle tertile vs highest tertile	2.35 (.34 - 16.27)	.39	1.25 (.29 - 5.45)	.77
Lowest tertile vs middle tertile	.32 (.05 - 2.02)	.22	.66 (.14 - 3.10)	.60
**School water consumption (0-3)**				
Lowest tertile vs highest tertile	-.34 (-.94 - .25)	.26	-.07 (-.68 - .54)	.82
Middle tertile vs highest tertile	-.12 (-.72 - .48)	.69	-.43 (-1.03 - .16)	.15
Lowest tertile vs middle tertile	-.22 (-.83 - .38)	.47	.36 (-.23 - .96)	.23
**PA-related parenting practices**				
**Sedentary (% per day at school)**				
Cluster 2 vs Cluster 1	.03 (-4.51 - 4.58)	.99	1.46 (-3.40 - 6.31)	.56
Cluster 3 vs Cluster 1	.91 (-7.84 - 9.66)	.84	-1.24 (-10.60 - 8.12)	.79
Cluster 4 vs Cluster 1	-.02 (-3.90 - 3.85)	.99	-.76 (-4.47 - 2.95)	.69
Cluster 3 vs Cluster 2	.88 (-7.91 - 9.66)	.84	-2.69 (-12.35 - 6.96)	.58
Cluster 4 vs Cluster 2	-.06 (-4.52 - 4.41)	.98	-2.22 (-7.06 - 2.62)	.37
Cluster 4 vs Cluster 3	-.93 (-9.42 - 7.55)	.83	.48 (-8.75 - 9.70)	.92
**Light PA (% per day at school)**				
Cluster 2 vs Cluster 1	.17 (-3.69 - 4.03)	.93	-.54 (-4.69 - 3.61)	.80
Cluster 3 vs Cluster 1	-.27 (-7.84 - 7.30)	.94	1.58 (-6.86 - 10.02)	.71
Cluster 4 vs Cluster 1	-.11 (-3.35 - 3.12)	.94	1.14 (-2.00 - 4.27)	.48
Cluster 3 vs Cluster 2	-.44 (-8.05 - 7.17)	.91	2.12 (-6.55 - 10.80)	.63
Cluster 4 vs Cluster 2	-.28 (-4.06 - 3.50)	.88	1.68 (-2.42 - 5.78)	.42
Cluster 4 vs Cluster 3	.16 (-7.20 - 7.51)	.97	-.44 (-8.76 - 7.88)	.92
**MVPA (% per day at school)**				
Cluster 2 vs Cluster 1	-.21 (-1.62 - 1.19)	.77	-.97 (-2.48 - .54)	.21
Cluster 3 vs Cluster 1	-.57 (-3.14 - 2.00)	.66	-.40 (-3.07 - 2.28)	.77
Cluster 4 vs Cluster 1	.13 (-1.08 - 1.33)	.84	-.39 (-1.56 - .77)	.51
Cluster 3 vs Cluster 2	-.35 (-2.96 - 2.25)	.79	.57 (-2.14 - 3.28)	.68
Cluster 4 vs Cluster 2	.34 (-1.02 - 1.70)	.62	.58 (-.90 - 2.06)	.44
Cluster 4 vs Cluster 3	.69 (-1.78 - 3.17)	.58	.01 (-2.62 - 2.63)	1.00
**Nutrition-related parenting practices**				
**Minimal two food types during lunch * (% yes)**				
Cluster 2 vs Cluster 1	1.69 (.17 - 16.81)	.65	1.28 (.21 - 7.91)	.79
Cluster 3 vs Cluster 1	.49 (.04 - 6.68)	.59	1.68 (.17 - 16.33)	.66
Cluster 4 vs Cluster 1	2.79 (.19 - 40.74)	.45	0.50 (.07 - 3.55)	.49

Cluster 3 vs Cluster 2	.29 (.02 - 3.94)	.35	1.31 (.13 - 13.15)	.82
Cluster 4 vs Cluster 2	1.65 (.11 - 24.37)	.71	.39 (.06 - 2.73)	.34
Cluster 4 vs Cluster 3	5.68 (.31 - 104.54)	.24	.30 (.03 - 3.21)	.32
**School water consumption (0-3)**				
Cluster 2 vs Cluster 1	.21 (-.42 - .85)	.51	.01 (-.66 - .67)	.98
Cluster 3 vs Cluster 1	-.15 (-.91 - .61)	.70	-.28 (-1.04 - .49)	.48
Cluster 4 vs Cluster 1	.06 (-.66 - .77)	.87	-.01 (-.77 - .74)	.98
Cluster 3 vs Cluster 2	-.37 (-1.17 - .43)	.37	-.29 (-1.07 - .49)	.47
Cluster 4 vs Cluster 2	-.16 (-.90 - .58)	.68	-.02 (-.77 - .73)	.96
Cluster 4 vs Cluster 3	.21 (-.67 - 1.09)	.64	.27 (-.59 - 1.12)	.54

** Binary outcome: Generalized estimating equation is used. Interaction term is Exp(B), which is the odds ratio of the first subgroup (e.g., boys) divided by the odds ratio of the second subgroup (e.g., girls), in which the odds ratio of the second group (girls) is the reference group.*
Significance level for the interaction-term: p<0.10.
Clusters nutrition-related parenting practices: Cluster 1 "high involvement and supportive"; Cluster 2 "low covert control and non-rewarding"; Cluster 3 "low involvement and indulgent"; Cluster 4 "high covert control and rewarding".
Clusters PA-related parenting practices: Cluster 1 "high involvement and supportive"; Cluster 2 "moderate involvement, indulgent of child's sedentary activities"; Cluster 3 "low involvement and indulgent"; Cluster 4 "moderate involvement, supportive of child's sedentary activities".
Abbreviations: HPSF: Healthy primary School of the Future; CI: confidence interval; p: p-value; ES: effect size; PA: physical activity; MVPA: moderate-to-vigorous physical activity; na: not applicable.

Table S2c. Pairwise comparisons of the effect modifiers of HPSF on intervention effects at home

	Full HPSF vs. control		Partial HPSF vs. control	
	B (95% C.I.)	p	B (95% C.I.)	p
**SES**				
**Sedentary (% per day at home)**				
Lowest tertile vs highest tertile	3.41 (-.03 - 6.84)	**.05**	3.85 (.49 - 7.22	**.02**
Middle tertile vs highest tertile	2.70 (-.68 - 6.09)	.12	1.97 (-1.41 - 5.34)	.25
Lowest tertile vs middle tertile	.70 (-2.84 - 4.25)	.70	1.89 (-1.54 - 5.32)	.28
**Light PA (% per day at home)**				
Lowest tertile vs highest tertile	-2.88 (-5.42 - -.34)	**.03**	-2.81 (-5.29 - -.32)	**.03**
Middle tertile vs highest tertile	-1.26 (-3.77 - 1.24)	.32	-1.31 (-3.81 - 1.18)	.30
Lowest tertile vs middle tertile	-1.62 (-4.23 - 1.00)	.23	-1.50 (-4.03 - 1.04)	.25
**MVPA (% per day at home)**				
Lowest tertile vs highest tertile	-.59 (-2.14 - .95)	.45	-1.10 (-2.61 - .41)	.15
Middle tertile vs highest tertile	-1.45 (-2.98 - .07)	**.06**	-.68 (-2.20 - .83)	.38
Lowest tertile vs middle tertile	.86 (-0.73 - 2.46)	.29	-.42 (-1.96 - 1.12)	.59
**Minimal two food types during breakfast * (% yes)**				
Lowest tertile vs highest tertile	3.22 (1.44 - 7.21)	.15	3.24 (1.51 - 6.97)	.12
Middle tertile vs highest tertile	3.24 (1.53 - 6.87)	.12	1.31 (.61 - 2.82)	.73
Lowest tertile vs middle tertile	.99 (.44 - 2.26)	.99	2.48 (1.15 - 5.33)	.24
**PA-related parenting practices**				
**Sedentary (% per day at home)**				
Cluster 2 vs Cluster 1	1.10 (-2.80 - 5.01)	.58	.15 (-3.97 - 4.27)	.94
Cluster 3 vs Cluster 1	3.21 (-2.88 - 9.30)	.30	.21 (-5.71 - 6.14)	.94
Cluster 4 vs Cluster 1	1.52 (-2.29 - 5.34)	.43	.52 (-3.01 - 4.06)	.77
Cluster 3 vs Cluster 2	2.11 (-4.00 - 8.22)	.50	.06 (-6.08 - 6.20)	.98
Cluster 4 vs Cluster 2	.42 (-3.43 - 4.27)	.83	.37 (-3.59 - 4.33)	.85
Cluster 4 vs Cluster 3	-1.69 (-7.63 - 4.25)	.58	.31 (-5.38 - 6.00)	.91
**Light PA (% per day at home)**				
Cluster 2 vs Cluster 1	-.43 (-3.33 - 2.46)	.77	.46 (-2.56 - 3.48)	.77
Cluster 3 vs Cluster 1	-2.15 (-6.61 - 2.32)	.34	-.08 (-4.61 - 4.45)	.97
Cluster 4 vs Cluster 1	-.48 (-3.23 - 2.27)	.73	-.61 (-3.24 - 2.01)	.65
Cluster 3 vs Cluster 2	-1.71 (-6.19 - 2.76)	.45	-.54 (-5.33 - 4.26)	.83
Cluster 4 vs Cluster 2	-.04 (-2.87 - 2.78)	.98	-1.07 (-4.00 - 1.86)	.47
Cluster 4 vs Cluster 3	1.67 (-2.71 - 6.04)	.45	-.54 (-4.98 - 3.91)	.81
**MVPA (% per day at home)**				
Cluster 2 vs Cluster 1	-.67 (-2.43 - 1.09)	.45	-.59 (-2.43 - 1.25)	.53
Cluster 3 vs Cluster 1	-1.07 (-3.77 - 1.63)	.44	-.15 (-2.70 - 2.40)	.91
Cluster 4 vs Cluster 1	-1.06 (-2.73 - .61)	.21	.08 (-1.44 - 1.59)	.92
Cluster 3 vs Cluster 2	-.40 (-3.21 - 2.41)	.78	.44 (-2.21 - 3.09)	.74

Cluster 4 vs Cluster 2	-.38 (-2.18 - 1.41)	.67	.66 (-1.12 - 2.44)	.46
Cluster 4 vs Cluster 3	.01 (-2.59 - 2.62)	.99	.22 (-2.22 - 2.67)	.86
**Nutrition-related parenting practices**				
**Minimal two food types during breakfast * (% yes)**				
Cluster 2 vs Cluster 1	.51 (.08 - 3.08)	.46	1.06 (.18 - 6.17)	.94
Cluster 3 vs Cluster 1	1.23 (.11 - 14.10)	.87	1.32 (.14 - 12.77)	.81
Cluster 4 vs Cluster 1	.50 (.06 - 4.08)	.51	2.40 (.35 - 16.55)	.37
Cluster 3 vs Cluster 2	2.41 (0.24 - 24.77)	.46	1.24 (.14 - 11.08)	.85
Cluster 4 vs Cluster 2	.98 (.14 - 7.00)	.98	2.25 (.37 - 13.72)	.38
Cluster 4 vs Cluster 3	.40 (.03 - 5.81)	.50	1.82 (.18 - 18.16)	.61

** Binary outcome: Generalized estimating equation is used. Interaction term is Exp(B), which is the odds ratio of the first subgroup (e.g., boys) divided by the odds ratio of the second subgroup (e.g., girls), in which the odds ratio of the second group (girls) is the reference group.*

Significance level for the interaction-term: p<0.10.

Clusters nutrition-related parenting practices: Cluster 1 "high involvement and supportive"; Cluster 2 "low covert control and non-rewarding"; Cluster 3 "low involvement and indulgent"; Cluster 4 "high covert control and rewarding".

Clusters PA-related parenting practices: Cluster 1 "high involvement and supportive"; Cluster 2 "moderate involvement, indulgent of child's sedentary activities"; Cluster 3 "low involvement and indulgent"; Cluster 4 "moderate involvement, supportive of child's sedentary activities".

Abbreviations: HPSF: Healthy primary School of the Future; CI: confidence interval; p: p-value; ES: effect size; PA: physical activity; MVPA: moderate-to-vigorous physical activity; na: not applicable.
